# Neuropsychiatric-pharmacologic synergy: interaction between psychiatric polypharmacy and cancer treatment-related neurotoxicity

**DOI:** 10.1093/omcr/omag107

**Published:** 2026-06-21

**Authors:** Sean P Woytowitz, Charles Manchee

**Affiliations:** Department of Psychiatry and the Behavioral Sciences, Keck School of Medicine of USC, 1975 Zonal Ave, Los Angeles, CA 90033, United States of America; Department of Psychiatry and the Behavioral Sciences, Keck Medicine of USC, 1500 San Pablo St, Los Angeles, CA 90033, United States of America

**Keywords:** altered mental status, delirium, polypharmacy, psycho-oncology, stem cell transplant

## Abstract

Altered mental status (AMS) in oncology populations frequently reflects complex interactions among medical, pharmacologic, and neuropsychiatric factors. Accurate identification of the primary contributors is crucial to minimize morbidity. We report the case of a man in his 60s with a relapsed plasma cell malignancy, status post autologous stem-cell transplant, who experienced recurrent AMS during ongoing combination chemotherapy and psychiatric polypharmacy. Neuroimaging excluded structural or vascular lesions. Laboratory evaluation revealed intermittent mild renal dysfunction without metabolic crisis. His medication regimen included multiple serotonergic, dopaminergic, and sedative agents; clinical improvement followed medication rationalization. This case highlights the multifactorial nature of AMS in cancer survivors, in which post-transplant neuroinflammation, systemic metabolic stress, and overlapping drug effects create a brain vulnerable to toxic-metabolic encephalopathy. A systems-based, interdisciplinary approach is essential for diagnosing and preventing AMS in post-transplant oncology patients. Early recognition and medication simplification can reduce recurrence and enhance cognitive function.

## Introduction

Altered mental status (AMS) is an increasingly recognized complication of cancer therapy, particularly among patients undergoing hematopoietic stem-cell transplantation (HSCT). Post-transplant survivors frequently experience neurocognitive deficits arising from inflammation, fatigue, sleep disturbance, and blood–brain barrier disruption, creating vulnerability to subsequent neuropsychiatric toxicity [1]. Likewise, cancer-related cognitive impairment involves cytokine activation, oxidative stress, and neuronal changes that sensitize the central nervous system to additional pharmacologic insults [2].

Turossi-Amorim et al. documented that psychotropics can interact pharmacodynamically with chemotherapeutic agents, increasing the risk of toxicity [3]. Following autologous stem-cell transplant, Otsuki et al. demonstrated that delirium occurs in a significant proportion of patients, with multiple predisposing and precipitating factors contributing simultaneously [4], reinforcing that even small stressors such as overlapping psychoactive drugs may trigger acute encephalopathy. Beyond individual drug effects, polypharmacy itself appears to worsen delirium trajectories. Reisinger et al. found that higher medication burden correlates with persistence and severity of delirium, suggesting that psychiatric polypharmacy is a modifiable risk factor [5].

Yet, interventions to mitigate these risks remain underdeveloped, particularly within medically complex populations such as the one discussed here [6]. The present case highlights the importance of systematic medication reconciliation as a preventive strategy for encephalopathy in post-transplant oncology patients.

## Case presentation

We present a man in his 60s with a relapsed plasma cell malignancy and longstanding psychiatric history including depression, anxiety, PTSD, and remote polysubstance use involving alcohol, benzodiazepines, and cocaine. He was initially diagnosed with multiple myeloma in October 2020 after presenting with right-sided pain and was found to have lytic lesions throughout the axial skeleton. Diagnostic evaluation demonstrated hypermetabolic osseous lesions on PET imaging and a bone marrow biopsy confirmed the diagnosis of multiple myeloma. He underwent eight cycles of VRd (triple therapy consisting of bortezomib, lenalidomide, and dexamethasone) with subsequent remission, followed by high-dose melphalan and autologous stem-cell transplantation (ASCT) in October 2021.

During transplant hospitalization in 2021, psychiatry evaluated him for anxiety, hopelessness, and insomnia related to the stress of diagnosis and treatment. He reported chronic mood and anxiety symptoms but maintained organized thought processes and intact cognition. He denied any familial psychiatric history at this time. No significant neuropsychiatric complications occurred during the transplant course.

Approximately three years post-transplant, in early 2024, he developed progressive fatigue, dizziness, and anemia requiring transfusion. Bone marrow biopsy demonstrated disease relapse. He was treated initially with DPd (daratumumab, pomalidomide, and dexamethasone) and later with Isa-Pd (isatuximab, pomalidomide, and dexamethasone). These regimens were complicated by gastrointestinal toxicity, intermittent infections, and fluctuating renal function. Throughout this period, he remained on a complex daily outpatient psychotropic regimen including olanzapine 30 mg, sertraline 200 mg, duloxetine 120 mg, mirtazapine 15 mg, ropinirole 0.5 mg, clonazepam 1 mg, levetiracetam, and chronic opioids for pain.

In early 2025, he began experiencing recurrent episodes of slowed speech, light-headedness, tremor, and confusion. He and his wife noted progressive psychomotor slowing, handwriting changes, memory lapses, and restless legs over several months. Laboratory studies during these episodes demonstrated anemia and intermittent mild acute kidney injury without major metabolic derangements. No changes to his Isa-Pd chemotherapeutic regimen were made at this time.

**Figure 1:**
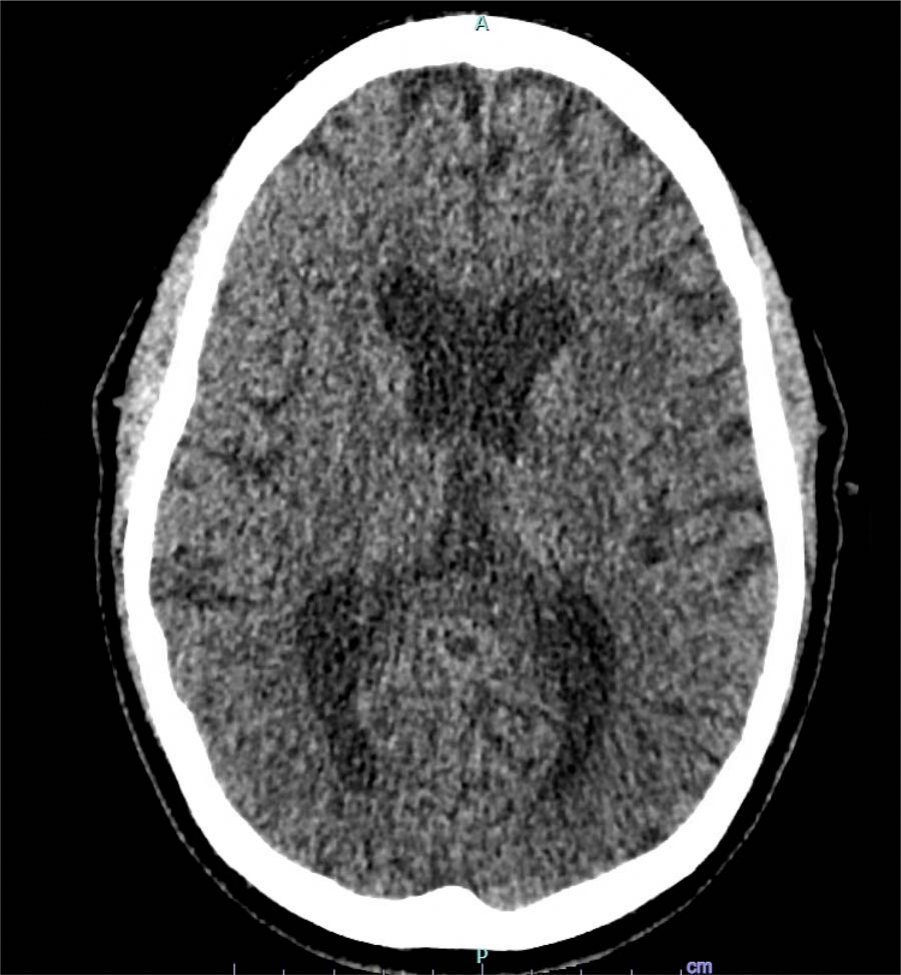
Computed-tomography (CT) of the head from 04/03/2025 demonstrating no evidence of hemorrhage, infarct, or large-vessel occlusion.

**Figure 2:**
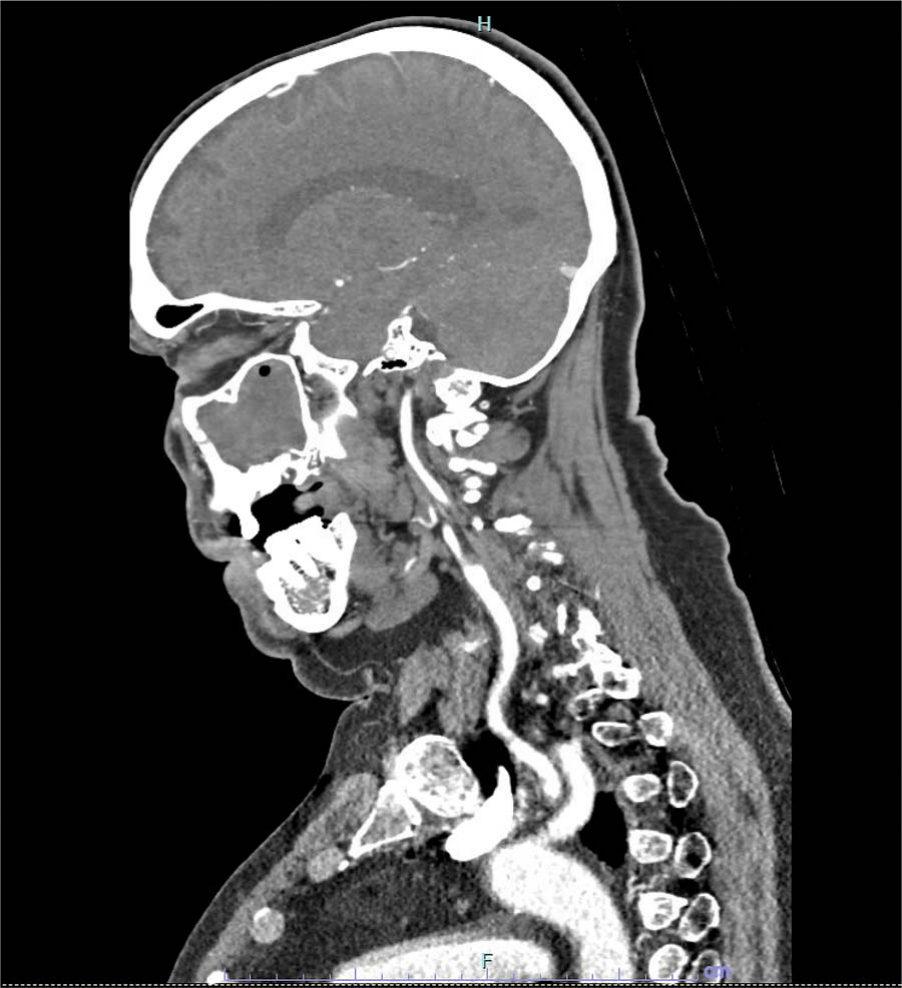
Computed-tomography-angiography (CTA) of the head and neck demonstrating no evidence of hemorrhage, infarct, or large-vessel occlusion.

On April 3, 2025, he presented to an outpatient oncology clinic with marked speech latency, subjective presyncope, and slowed responsiveness. A code stroke was activated; however, CT head and CT angiography of the head and neck demonstrated no hemorrhage, infarct, or large-vessel occlusion (Figs 1 and 2). His presentation was felt to be consistent with metabolic or medication-related encephalopathy.

Psychiatry was consulted the same day for evaluation of encephalopathy in the setting of polypharmacy. Examination revealed slow and effortful speech, cognitive inefficiency, and a mild tremor. Although he was taking multiple serotonergic agents—including sertraline, duloxetine, mirtazapine, meperidine, and ondansetron—he lacked autonomic instability, hyperreflexia, or clonus and serotonin syndrome was deemed unlikely. Still, psychiatry recommended minimizing serotonergic polypharmacy.

Collateral from his outpatient psychiatrist revealed longstanding reliance on high-dose olanzapine due to prior episodes of paranoia and suicidality during taper attempts. The combination of dopaminergic and antidopaminergic agents raised concern for extrapyramidal symptoms (EPS) and drug-induced parkinsonism. The decision was made following the first psychiatric evaluation of the patient to reduce the patient’s psychiatric medication burden from the regimen listed above to only levetiracetam 1500 mg, ropinirole 0.5 mg, sertraline 100 mg and mirtazapine 15 mg daily while inpatient, with discontinuation of olanzapine, duloxetine, and clonazepam.

Over subsequent days, symptoms fluctuated. Neurologic examination demonstrated mild cogwheel rigidity of the right elbow, moderate-frequency low-amplitude tremor, and continued speech latency. He described chronic restless legs and fragmented sleep, raising concern for a parkinsonian spectrum disorder. However, neither neurology nor psychiatry identified autonomic changes, hallucinations, or REM sleep behavior disorder, making olanzapine-induced EPS more likely.

Neurology and psychiatry jointly diagnosed multifactorial delirium superimposed on chronic ASCT-related cognitive vulnerability. Contributors included polypharmacy with serotonergic, dopaminergic, sedative, and antidopaminergic agents; intermittent AKI impairing drug clearance; chemotherapy- and transplant-related neurotoxicity; anemia and systemic physiologic stress; and possible mild EPS. Posterior reversible encephalopathy syndrome (PRES) was considered but felt unlikely given persistently normal imaging.

Following systematic deprescribing, the patient demonstrated marked improvement in cognition, gait, and tremor. His mood remained stable, and he expressed relief at improved cognitive function. At discharge, he was maintained only on sertraline 100 mg, ropinirole 0.5 mg, and mirtazapine 15 mg daily, with plans for outpatient follow-up with psychiatry and neurology. Further documentation post-discharge indicated no changes to this simplified psychiatric medication regimen, though a few months later the patient unfortunately expired secondary to progression of his cancer.

## Discussion

This case illustrates the complex neuropsychiatric–pharmacologic interactions that can precipitate AMS in post-transplant oncology populations. Although this patient did not receive an inherently neurotoxic chemotherapy regimen, his recurrent encephalopathy emerged from the intersection of post-ASCT brain vulnerability, systemic physiologic stressors, and psychiatric polypharmacy. This trajectory demonstrates that in cancer survivors—particularly those with prior hematopoietic stem-cell transplantation—even moderate psychoactive medication burden can produce disproportionate neurologic effects.

HSCT is increasingly recognized to produce persistent neurocognitive deficits due to neuroinflammation, fatigue, sleep disruption, and blood–brain barrier alterations [1]. These changes establish a chronically sensitized CNS prone to toxic-metabolic encephalopathy when exposed to pharmacologic or metabolic stress. Up to 39% of autologous HSCT recipients develop delirium or encephalopathy within 30 days post-transplant, commonly from multifactorial causes including medication effects, anemia, infection, or organ dysfunction [7]. Although this patient’s transplant occurred several years earlier, his chronic sleep disturbance, fatigue, and cognitive slowing were consistent with long-term HSCT survivorship effects.

Unlike regimens known for significant CNS toxicity (e.g. high-dose cytarabine, intrathecal chemotherapy, platinum agents), the therapies used in this patient have minimal direct central neurotoxic potential. Bortezomib primarily causes peripheral neuropathy, with central effects occurring in < 2% of cases and usually in the context of systemic stressors [8]. Immunomodulators such as lenalidomide commonly cause fatigue and cognitive fog, while frank encephalopathy remains rare [9]. High-dose melphalan used in ASCT conditioning carries a < 1% risk of encephalopathy, largely in the setting of renal impairment [10]. Thus, neither this patient’s induction nor relapse regimens would be expected to be the primary drivers of his recurrent encephalopathy. Nonetheless, chemotherapy-related cognitive impairment remains common and is associated with deficits in working memory, executive function, and processing speed [11], likely lowering this patient’s delirium threshold.

The strongest contributors to this patient’s recurrent AMS were pharmacologic. He was simultaneously prescribed high-dose olanzapine, sertraline, duloxetine, mirtazapine, clonazepam, levetiracetam, and chronic opioids—representing overlapping serotonergic, dopaminergic, GABAergic, and sedative agents. Chemotherapeutic exposure, systemic inflammation, and intermittent renal dysfunction may further amplify these effects by altering blood–brain barrier permeability and drug metabolism, increasing central nervous system exposure to psychoactive medications and lowering the threshold for toxic-metabolic encephalopathy. Toxic-metabolic encephalopathy associated with polypharmacy is well documented, particularly when serotonergic medications or serotonergic antiemetics are combined [12]. His regimen placed him at risk for subacute serotonin toxicity, which can manifest with tremor, tachycardia, and cognitive change even in the absence of full serotonin syndrome [13].

Chronic dopamine blockade from olanzapine likely contributed to his tremor, cogwheel rigidity, and bradykinesia. Drug-induced parkinsonism is among the most common extrapyramidal syndromes in older adults, with heightened risk following chemotherapy or transplant-related neuroinflammation [14]. The improvement in motor symptoms after olanzapine discontinuation further supports this mechanism.

Benzodiazepines and opioids, both used chronically, are well-established deliriogenic agents, with withdrawal or intermittent dosing precipitating sleep fragmentation, anxiety, and autonomic instability [15]. Episodes of encephalopathy coincided with anemia, infections, dehydration, and mild acute kidney injury—each independently capable of precipitating delirium in immunocompromised patients [7]. AKI further impaired clearance of renally excreted medications, amplifying CNS drug exposure.

In similar cases, diagnosis should proceed as a structured exclusion process: rule out primary neurologic causes (neuroimaging, seizure activity, focal deficits); exclude infectious etiologies; assess metabolic contributors; review medication burden with attention to recent changes, drug–drug interactions, and renally cleared agents; and evaluate for withdrawal states or subclinical toxicity (e.g. serotonin excess). Improvement with targeted deprescribing further supports a toxic-metabolic etiology.

This case demonstrates how CNS vulnerability, polypharmacy, and systemic stressors interact synergistically to produce encephalopathy. AMS improved reliably with medication simplification, underscoring the central role of pharmacologic burden. The novelty of this case is found in its demonstration of late post-transplant neuropsychiatric vulnerability and exploration of our developing understanding of psychiatric polypharmacy. Our findings highlight the need for proactive medication rationalization and interdisciplinary coordination between oncology and psychiatry to reduce morbidity in post-transplant cancer survivors. We support a collaborative effort between primary and consulting teams, both inpatient and outpatient, in the development of medication regimens to avoid polypharmacy and combined toxicities to prevent cases such as this; we also recognize that the quantified risk of multifactorial mental status changes such as the one demonstrated here or the existence of a causal relationship between our proposed mechanism of AMS and the patient’s mental status changes can’t be assessed by this study.

## Conclusion

This case highlights the profound neuropsychiatric vulnerability that can emerge at the intersection of cancer therapy and extensive psychoactive medication exposure. Despite the absence of a highly neurotoxic chemotherapy regimen, multifactorial cognitive sensitivity and polypharmacy produced recurrent toxic-metabolic encephalopathy. Systematic medication rationalization led to marked cognitive and functional improvement. These findings emphasize the central importance of proactive psychopharmacologic oversight and close interdisciplinary collaboration to prevent recurrent altered mental status in medically complex oncology patients.

## Consent

The case described below documents the medical course of a patient that has since passed away. Analysis of his case began following his passing. Meaningful attempts to contact next-of-kin were made using all available contact information provided by the patient to the hospital, but contact could not be made. For this reason, the following case study lacks an explicit consent form; however, all patient identifying information has been removed to the best of the authors’ ability without infringing on the scientific value of the manuscript. Anonymization has followed the Committee on Publication Ethics (COPE) and ICMJE recommendations as well as HIPAA ‘Safe Harbor’ deidentification.
